# Automatic detection of optic canal fractures and recognition and segmentation of anatomical structures in the orbital apex based on artificial intelligence

**DOI:** 10.3389/fcell.2025.1609028

**Published:** 2025-05-30

**Authors:** Yu-Lin Li, Yu-Hao Li, Mu-Yang Wei, Guang-Yu Li

**Affiliations:** ^1^ Department of Ophthalmology, The Second Norman Bethune Hospital of Jilin University, Changchun, China; ^2^ International School, Beijing University of Posts and Telecommunications, Bei Jing, China

**Keywords:** optic canal fractures, CT, enhanced orbital CT, deep learning, AI, YOLOv7, UNET

## Abstract

**Background and objectives:**

Traumatic optic neuropathy (TON) caused by optic canal fractures (OCF) can result in severe visual impairment, even blindness. Timely and accurate diagnosis and treatment are crucial for preserving visual function. However, diagnosing OCF can be challenging for inexperienced clinicians due to atypical OCF changes in imaging studies and variability in optic canal anatomy. This study aimed to develop an artificial intelligence (AI) image recognition system for OCF to assist in diagnosing OCF and segmenting important anatomical structures in the orbital apex.

**Methods:**

Using the YOLOv7 neural network, we implemented OCF localization and assessment in CT images. To achieve more accurate segmentation of key anatomical structures, such as the internal carotid artery, cavernous sinus, and optic canal, we introduced Selective Kernel Convolution and Transformer encoder modules into the original UNet structure.

**Results:**

The YOLOv7 model achieved an overall precision of 79.5%, recall of 74.3%, F1 score of 76.8%, and mAP@0.5 of 80.2% in OCF detection. For segmentation tasks, the improved UNet model achieved a mean Intersection over Union (mIoU) of 92.76% and a mean Dice coefficient (mDice) of 90.19%, significantly outperforming the original UNet. Assisted by AI, ophthalmology residents improved their diagnostic AUC-ROC from 0.576 to 0.795 and significantly reduced diagnostic time.

**Conclusion:**

This study developed an AI-based system for the diagnosis and treatment of optic canal fractures. The system not only enhanced diagnostic accuracy and reduced surgical collateral damage but also laid a solid foundation for the continuous development of future intelligent surgical robots and advanced smart healthcare systems.

## Graphical Abstract

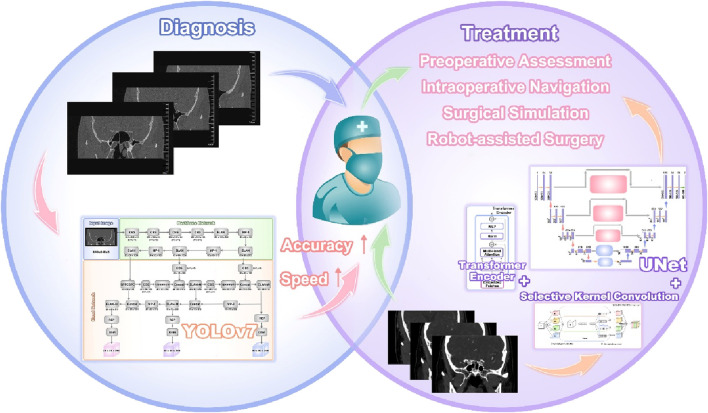

## 1 Introduction

The optic canal is formed by the upper and lower wings of the sphenoid bone, the outer side of the sphenoid body, and the outer bone wall of the posterior ethmoid sinus (sphenoid sinus). Serving as the conduit for the optic nerve and ophthalmic artery to enter the orbit from the skull, it plays a crucial role in vision. Optic canal fractures (OCF) typically stems from trauma to the orbit or face, including scenarios such as blunt trauma, car accidents, falls, or adjacent skull fractures. When external impact forces are transmitted to the optic nerve through the surrounding bone structure, or when fractured fragments directly compress or puncture the optic nerve, it can result in traumatic optic neuropathy (TON), leading to partial or complete loss of visual function (Hokazono et al., 2019; Steinsapir and Goldberg, 2011). The pathophysiological mechanisms of TON are highly intricate and have not been completely elucidated to date. When the optic nerve undergoes mechanical shearing forces, the axons of Retinal Ganglion Cells (RGC) and the vessels supplying the nerves are disrupted, resulting in axonal degeneration of RGC. Subsequently, this triggers a cascade of secondary injuries such as oxidative stress, inflammation, cell apoptosis, release of kinins, and demyelination of axons. These successive damages further exacerbate the injury to RGC, ultimately leading to visual impairment and loss (Lin et al., 2021). Research indicates that approximately 50% of TON patients will experience permanent loss of vision (Reddy et al., 2015).

Imaging examinations play a crucial role in the diagnosis of TON, particularly with the indispensable use of computed tomography (CT) and thin-section scans of the optic canal. These examinations contribute to a comprehensive assessment of the extent of fractures and potential optic nerve damage, providing vital references for accurate diagnosis and subsequent treatment strategies. However, due to the optic canal’s characteristics, including its deep location, narrow diameter, thin bony walls, small volume, and complexity of adjacent bony structures, the subtle changes in imaging for OCF can be elusive. Existing methods, such as conventional CT imaging, often fail to detect displaced fractures or deformities in the optic canal, leading to missed diagnoses. Moreover, current segmentation and detection techniques are limited in their ability to accurately identify and segment critical anatomical structures surrounding the optic canal, such as the internal carotid artery, cavernous sinus, and optic nerve. Displaced fractures or deformities of the optic canal and its anatomical structures are often challenging to detect. Even in the presence of apparent clinical symptoms, such as visual impairment or loss, imaging examinations may only reveal subtle fractures in the bones (Nagasao et al., 2018). Therefore, for less experienced resident physicians, accurately and rapidly diagnosing OCF remains a challenging task in clinical practice.

The optic canal decompression surgery is one of the crucial therapeutic approaches for TON. Its principle lies in alleviating the mechanical compression of the visual pathway by removing the surrounding bony structures, thereby reducing intraneural pressure and restoring local blood circulation to prevent optic nerve damage (Li and Guo, 2022). Research indicates that surgical intervention within 7 days after TON significantly lowers the long-term risk of visual impairment. Conversely, TON patients without timely intervention may experience reduced central retinal artery blood flow and a significant decrease in the thickness of the retinal nerve fiber layer, resulting in a poorer prognosis (Lin et al., 2021; Tu et al., 2023; Peng et al., 2011; Li and Guo, 2022; Tu et al., 2023). Therefore, promptly determining the extent and severity of OCF and implementing surgery is crucial for visual recovery. In recent years, with continuous improvements in microsurgical techniques and instruments, endoscopic transethmoidal optic canal decompression (ETOCD) has emerged as the current mainstream surgical method. Its advantages include a broad field of view, minimal damage, and rapid recovery (Liu et al., 2023). Retrospective studies suggest that TON patients undergoing ETOCD exhibit better long-term outcomes compared to conservative treatment (Horiguchi et al., 2010; Ma et al., 2018; Yu et al., 2016; Yan et al., 2017).

However, due to the intricate anatomy of the orbital apex, the diagnosis and surgical treatment of diseases involving this critical region are often challenging (Lin et al., 2021). The orbital apex serves as a bony tunnel through which many crucial neurovascular structures enter the eye socket from the cranial cavity. It houses cranial nerves III, IV, and VI, the ophthalmic division of the trigeminal nerve (V1), as well as the superior ophthalmic vein. The optic canal is situated above the orbital fissure, with its inner wall exhibiting significant variation due to the degree of pneumatization of the sphenoid sinus (Dallan et al., 2013; Trevino-Gonzalez et al., 2023). The internal carotid artery runs adjacent to the lateral wall of the sphenoid sinus and is a primary blood supply source to the eye socket. During its course through the bony canal in the temporal bone’s petrous part, it closely approaches the optic canal (Asal et al., 2019). In this confined anatomical region connecting the eye socket and the intracranial space, critical structures are often only millimeters apart. Inaccurate diagnosis or surgical procedures may result in compromised visual function and severe neurovascular complications (Abuzayed et al., 2009). Therefore, precise identification and assessment of the anatomical structures related to the sphenoid sinus, internal carotid artery, and optic canal are crucial to avoid potentially fatal complications such as internal carotid artery bleeding during surgery (Lin et al., 2021).

The primary goal of Artificial Intelligence (AI) is to develop models and algorithms capable of simulating human intelligence, enabling machines to engage in activities such as learning, reasoning, problem-solving, and decision-making. This encompasses various technologies, including machine learning, neural networks, and computer vision. In recent years, the application of deep learning in accomplishing medical image recognition tasks has become a research hotspot. Various Convolutional Neural Networks (CNNs) have demonstrated remarkable performance in tasks such as image classification, object detection, and semantic segmentation (Zhang et al., 2023). Currently, image recognition systems based on Convolutional Neural Networks (CNNs) find widespread applications in the diagnosis and treatment of ophthalmic diseases. These systems assist in the precise analysis of digitized eye CT and MRI images, fundus images, and Optical Coherence Tomography (OCT) scan images to aid in the diagnosis and formulation of surgical plans for ophthalmic conditions. This includes diseases such as ocular tumors, diabetic retinopathy, glaucoma, and macular degeneration (Leong et al., 2022; Zhao, 2018; Ahuja et al., 2022; Nanegrungsunk et al., 2022; Ting et al., 2019; Bi et al., 2019; Xu et al., 2023a; Zhu et al., 2023). In the realm of orbital diseases, there are currently AI-assisted diagnostic models tailored for orbital bone fractures, thyroid-related eye diseases, and intraorbital tumors. Additionally, there are automatic segmentation models specifically designed for the orbital region (Li et al., 2020; Song et al., 2021; Hamwood et al., 2021). However, there is currently a lack of automatic segmentation models specifically designed for the anatomical structures of the orbital apex, and there is also a deficiency in artificial intelligence models that can assist in diagnosing optic canal fractures. It is worth noting that CNN-based image recognition methods still have limitations in medical image segmentation and may not fully meet the requirements for accurate segmentation. Due to the limited size of the receptive field of convolutional kernels, many standard convolutional neural networks face challenges in capturing comprehensive contextual information, especially when dealing with complex relationships in medical images. Additionally, due to constraints in the number of samples within medical image datasets, achieving satisfactory accuracy in medical image segmentation tasks can often be challenging (Zhang et al., 2023; ST-Unet et al., 2023; Yamashita et al., 2018).

This study has developed an intelligent diagnosis and treatment system for optic canal fractures (OCF), which combines a YOLOv7-based model for detecting optic canal fractures with a novel UNet-based network for recognizing anatomical structures in the orbital apex. These components together form a comprehensive OCF diagnosis and treatment system. Not only does this system serve as a vital tool for assisting in the diagnosis of OCF, but it also establishes a foundation for the safety assessment of optic canal decompression surgeries. Furthermore, the key issue addressed by this study is the significant challenges posed by existing diagnostic and segmentation methods, which often struggle to accurately detect optic canal fractures and segment the complex anatomical structures surrounding the optic canal. Current imaging techniques and manual segmentation methods are often imprecise or heavily reliant on the operator’s expertise, resulting in inconsistent outcomes. This study aims to overcome these limitations by developing an AI-driven system that automates these processes, providing clinicians with more accurate, efficient, and reliable diagnostics, ultimately improving patient outcomes and facilitating timely surgical interventions.

## 2 Materials and methods

This study adheres to the principles outlined in the Helsinki Declaration and has obtained approval from the Ethics Review Committee of the Second Norman Bethune Hospital of Jilin University. In this research, the review committee waived the requirement for informed consent.

### 2.1 Database

This study collected CT images of patients who underwent parallel optic canal CT and enhanced orbital CT examinations at the Second Norman Bethune Hospital of Jilin University China, from September 2020 to September 2022, forming two separate datasets. All patients were of Asian descent and were adults. Inclusion criteria for optic canal fractures patients included confirmation of optic canal fractures by radiologists and ophthalmologists, and undergoing ETOCD. For each patient, 2–6 high-quality consecutive CT scan images were selected from the CT image sequence. Table 1 shows the baseline demographic characteristics, and Table 2 displays the preoperative visual acuity of the patients.

**TABLE 1:** Baseline demographic characteristics of each group.

Demographic	Fracture	Non-fracture
Number of patients	93	47
Male	65	20
Female	28	27
Age, in years		
Mean ± standard deviation	45.02 ± 14.92	43.5 ± 14.69
Range	20–79	18–80

**TABLE 2:** Preoperative visual acuity of the patients.

Visual acuity category	Number of patients
NLP	8
LP	32
HM	23
FC	17
0.02–0.1	13

NLP: no light perception; LP: light perception; HM: hand motion; FC: finger counting; 0.02–0.1: corresponding standard logarithmic visual acuity chart.

#### 2.1.1 Optic canal CT image database

We selected a total of 652 optic canal CT images to form Dataset 1, used for training and testing optic canal fractures detection. The optic canal CT database includes a total of 509^−ΔΔCT^ images with signs of optic canal fractures (comprising 185 images with fractures only in the left eye, 207 images with fractures only in the right eye, and 117 images with fractures in both eyes). Additionally, 143 images showed no signs of optic canal fractures on either side. On high-resolution optic canal CT scans, direct signs of optic canal fractures include discontinuity, displacement, and/or fragmentation of the canal wall. Indirect signs may include fluid (hemorrhage), or air accumulation within the sphenoid or ethmoid sinuses. Fractures involving the ethmoid or sphenoid sinuses and orbital walls, as well as intraorbital fluid or air accumulation, may also be observed. Optic nerve damage may manifest as swelling, thickening, rupture, irregular thickening, or atrophy. The CT images with optic canal fractures in the dataset were independently assessed by three experienced radiologists. When consensus was reached on the diagnosis, bounding boxes were used to annotate the area of the optic canal containing the fracture. An additional senior physician was invited to adjust and correct the bounding boxes in cases where consensus was not reached among the three radiologists. The optic canal regions in the CT images were labeled using the online tool LabelImg and categorized into four classes: Fracture(R), Fracture(L), Non-fracture(R), Non-fracture(L). The dataset was split into training and testing sets in an 8:2 ratio. In this study, data augmentation techniques including resizing, random flipping, normalization, and padding were applied to the training set of Dataset 1.

#### 2.1.2 Enhanced orbital CT image database

For OCF patients undergoing ETOCD included in the study, we preoperatively evaluated the anatomy of the orbital apex using enhanced orbital CT scans. From these images, we selected a total of 200 enhanced orbital CT images to form Dataset 2, used for training and testing the segmentation of important anatomical structures in the orbital apex. We annotated three anatomical structures: the internal carotid artery (ICA), the optic canal (OC), and the sphenoid sinus (SS). The enhanced orbital CT images were independently assessed by three experienced radiologists. When consensus was reached on the diagnosis, the corresponding anatomical structures were annotated. An additional senior physician was invited to determine and annotate the corresponding anatomical structures in cases where consensus was not reached among the three radiologists. The LabelMe online tool was used to annotate the three anatomical structures in the orbital apex. The dataset was split into training and testing sets in an 8:2 ratio. We performed data augmentation on the original dataset, including geometric transformations, flipping, color space conversions, random cropping, random rotations, and introducing noise, to further increase the diversity of the training samples.

### 2.2 Network models and network training

**FIGURE 1:**
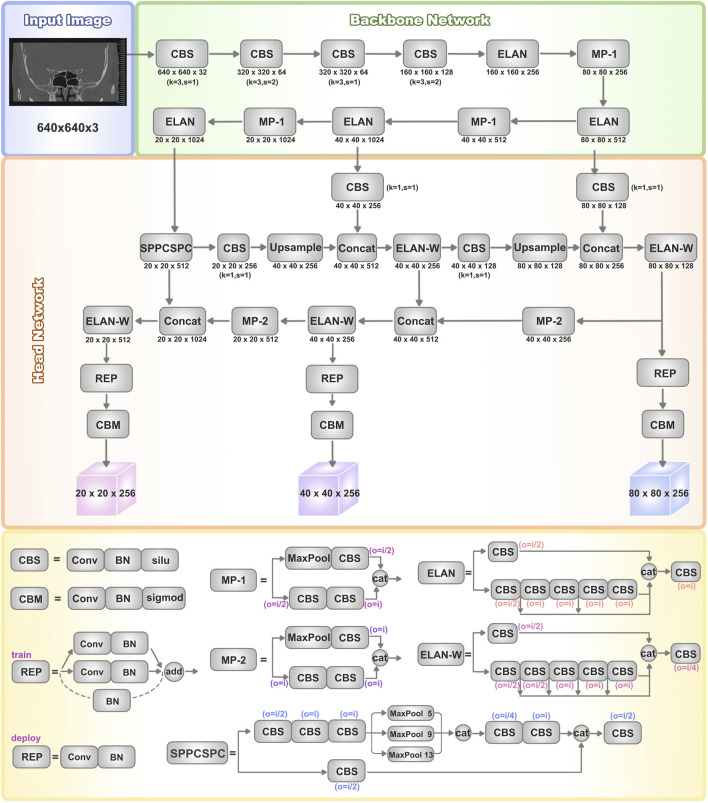
Architectures of YOLOv7. The basic structure of the YOLOv7 network includes the input, backbone network, and head network. It utilizes a convolutional neural network to simultaneously predict bounding boxes and class probabilities for all objects in an image. The backbone network consists of CBS, MP, and ELAN modules, which extract features from the input image and gradually reduce spatial dimensions while increasing channel numbers through convolutional operations for further processing. The head network consists of a series of convolutional layers, fully connected layers, and activation functions, used to predict the class, position, and confidence of the target objects. The head layer network outputs three different-sized feature maps, which are processed through Rep (Repetition) and conv (Convolution) layers to ultimately generate the predictions for the targets.

The overall structure and modules of the YOLOv7 network are depicted in Figure 1. The architecture of the YOLOv7 model comprises input, backbone network, and head network components. Initially, the raw input images undergo preprocessing to standardize their dimensions to 640 × 640, facilitating more efficient feature extraction by the Backbone network. The Backbone serves as the primary feature extraction network of YOLOv7, initially processing the input images to extract features, which are then received and integrated by the head network. The Backbone network consists of CBS, MP, and ELAN modules. The CBS module enhances network learning capability through convolution operations, data normalization, and the utilization of the SiLU activation function. The ELAN module controls gradient paths to enable the network to capture more features effectively. The MP1 module downsamples through two branches and combines extracted features using cascading operations. These processes collectively enhance YOLOv7’s feature extraction ability, leading to improved overall efficiency and accuracy.

The head network employs a Feature Pyramid Network (FPN) structure to enhance feature extraction. Three effective feature layers obtained from the backbone section are fused within this network component to integrate feature information from different scales. Accurate object detection and localization heavily depend on the head network’s capability to consolidate features into a cohesive whole. The SPPCSPC module combines Spatial Pyramid Pooling (SPP) and Cross Stage Partial Connection (CSPC). SPP expands the receptive field, enabling the algorithm to adapt to images of varying resolutions. CSPC improves feature transmission and network efficiency, reducing computational complexity while enhancing speed and accuracy. ELAN-W is analogous to the ELAN module, differing in the selection of output channel numbers for the second branch. MP2 functions similarly to MP1 but with multiple output channels. The Rep structure is utilized to adjust the number of image channels in the output features.

The original architecture of UNet comprises two main components. The first segment entails feature extraction, where each layer consists of two consecutive 3 × 3 convolutions with ReLU activation, followed by a 2 × 2 max-pooling layer. The latter part encompasses the decoding stage and the upsampling process facilitated by 2 × 2 deconvolutions, resulting in a halving of the input channel count. At each upsampling iteration, it merges with an equal number of channels corresponding to the feature extraction phase.

**FIGURE 2:**
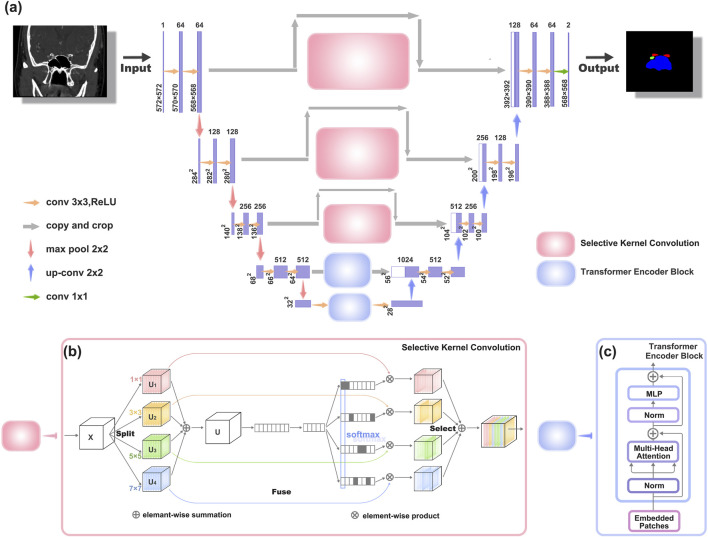
Architectures of the improved UNet network structure **(a)** The new network structure, which is an improvement upon the original UNet network. **(b)** Schematic diagram of the Selective Kernel Convolution (SK Conv) structure. SK Conv consists of Split, Fuse, and Select components. **(c)** Schematic diagram of the Transformer Encoder Block structure.

However, due to insufficient consideration for the semantic gap between the encoding and decoding stages, the original UNet structure exhibits certain limitations (Zhang et al., 2023). To address this issue and extract richer semantic information, we have introduced enhancements to the original UNet architecture, leading to the modified UNet network illustrated in Figure 2a.

To enhance texture representation within the UNet architecture (depicted in Figure 2b), we introduce a Texture Enhancement Module based on Selective Kernel Convolution (SKConv) within the first three skip connections. SKConv comprises three primary components: Split, Fuse, and Select. Split generates diverse paths by employing convolutional kernels of varying sizes, corresponding to different receptive field (RF) sizes of neurons. In our implementation, four branches of convolution kernels (1 × 1, 3 × 3, 5 × 5, and 7 × 7) were utilized. Subsequently, the Fuse operation amalgamates information from these diverse paths to derive a comprehensive and global representation, crucial for weight selection. Finally, Select combines feature maps of distinct kernel sizes based on selection weights. Through the integration of an attention mechanism within the convolutional kernel, SKConv enables adaptive alteration of neuron RF sizes during the inference phase.

Additionally, we incorporate a Semantic Perception Module, constructed using Transformer Encoder Block (TEB), within the last two skip connections of the UNet structure, addressing limitations associated with limited receptive fields (as illustrated in Figure 2c).

### 2.3 Performance assessments

#### 2.3.1 Object detection model evaluation metrics

After completing the training and testing phases of the model, it is essential to evaluate its performance using standardized metrics. For the object detection model used to assist in diagnosing OCF, we evaluate the performance of the YOLOv7 algorithm by calculating metrics such as true positives (TP), true negatives (TN), false positives (FP), and false negatives (FN). The evaluation metrics include precision, recall, mean average precision (mAP), precision-recall (PR) curve, and F1 score. Precision represents the proportion of samples predicted as positive that are actually positive, reflecting the model’s accuracy. Recall represents the proportion of actual positive samples predicted as positive, indicating the model’s ability to identify positive results. The F1 score is the harmonic mean of precision and recall, providing a comprehensive evaluation of the model by simultaneously considering precision and recall. A higher F1 score indicates a higher model quality. The formulas for calculating each evaluation metric are shown in Equations 1–3.

$$\text{Precision}=\frac{TP}{TP+FP}$$ (1)

$$\text{Recall}=\frac{TP}{TP+FN}$$ (2)

$$F1\text{ score}=\frac{2*\text{Precision}*\text{Recall}}{\text{Precision}+\text{Recall}}=\frac{2TP}{2TP+FP+FN}$$ (3)

Drawing the PR curve based on precision and recall values allows for a more comprehensive evaluation of the model. A larger area under the PR curve corresponds to higher average precision of the model. The Average Precision (AP) is the average precision value of the area under the PR curve and the coordinate axis. Mean Average Precision (mAP) is the average of AP values for each detected class. mAP@0.5 refers to the mAP calculated when the intersection over union (IoU) threshold is set to 0.5.

Furthermore, we utilize the Receiver Operating Characteristic (ROC) curve and the Area Under the Curve (AUC) of the ROC curve to evaluate the diagnostic capabilities of both ophthalmology residents and AI for OCF diagnosis.

#### 2.3.2 Semantic segmentation model evaluation metrics

We assess the similarity between the UNet model used for segmentation of orbital apex anatomical structures and manual segmentation using metrics such as Intersection over Union (IoU) and Dice coefficient. In this context, the intersection between the image segmented by the AI model and the manually segmented image constitutes the true positives (TP), the portion removed from the AI model’s segmented image constitutes false positives (FP), and the portion removed from the manually segmented image constitutes false negatives (FN). The formulas for calculation are provided in Equations 4, 5.

Dice scores and IoU values closer to 1 indicate that the AI model’s segmentation results are more similar to manual segmentation. mIoU represents the average IoU for each class, and mDice represents the average Dice score for each class. The formulas for calculation are provided in Equations 6, 7. Here, ‘k' denotes the total number of classes (in this study, there are three classes: optic canal, internal carotid artery, and sphenoid sinus).

$$\text{IoU}=\frac{TP}{TP+FP+FN}$$ (4)

$$\text{Dice}=\frac{2TP}{2TP+FP+FN}$$ (5)

$$\text{mIoU}=\frac{\sum_{i=1}^{k}IoUi}{k}$$ (6)

$$\text{mDice}=\frac{\sum_{i=1}^{k}\text{Dice}i}{k}$$ (7)

### 2.4 Statistical analysis

We conducted statistical analysis using GraphPad Prism 9.0 software and presented the data as mean ± standard error. To compare the differences in diagnostic capabilities and diagnostic time of ophthalmology residents before and after referencing AI, we employed paired-sample t-tests for analysis, considering differences with p < 0.05 to be statistically significant.

## 3 Results

### 3.1 Training and evaluation of YOLOv7 model for OCF recognition

**FIGURE 3:**
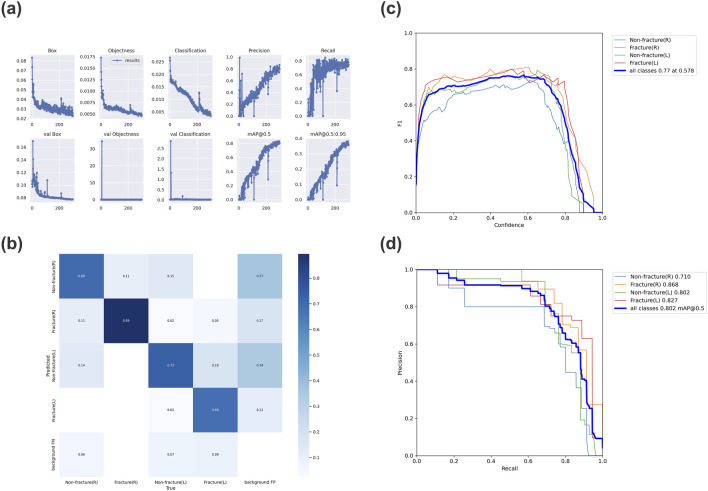
Evaluation results of the trained YOLOv7 model **(a)** Variation of each metric in YOLOv7 over 300 epochs. **(b)** Confusion matrix for the YOLOv7 model. **(c)** F1 curve for the YOLOv7 model, with an F1 score of 0.77. **(d)** Precision-recall curve for the YOLOv7 model, with an mAP_0.5 value of 0.802. (mAP_0.5: Average Precision, IoU = 0.5).

As the number of iterations increases, the loss value of YOLOv7 steadily decreases and eventually stabilizes, reaching its optimal performance after 300 training cycles (Figure 3a). We evaluated the model’s performance using precision, recall, mAP@0.5, and F1 score. The model demonstrates excellent detection performance for OCF. For detecting right-sided OCF, the precision, recall, mAP@0.5, and F1 score are 0.886, 0.739, 0.868, and 0.806, respectively. For left-sided OCF detection, the corresponding metrics are 0.75, 0.833, 0.827, and 0.789. The overall precision, recall, F1 score, and mAP@0.5 of the model are 0.795, 0.743, 0.768, and 0.802, respectively (Table 3).

**TABLE 3:** Evaluation metrics for YOLOv7 model performance.

Category	Precision	Recall	mAP@0.5	F1-score
All	0.795	0.743	0.802	0.768
Non-fracture(R)	0.74	0.686	0.71	0.712
Fracture(R)	0.886	0.739	0.868	0.806
Non-fracture(L)	0.804	0.714	0.802	0.756
Fracture(L)	0.75	0.833	0.827	0.789

Furthermore, the confusion matrix indicates that most targets are correctly predicted, demonstrating the model’s robust classification performance (Figure 3b). The F1 curve and PR curve of the model are shown in Figures 3c,d, confirming the significant reliability of the AI model in identifying OCF based on optic canal CT images post-training.

**FIGURE 4:**
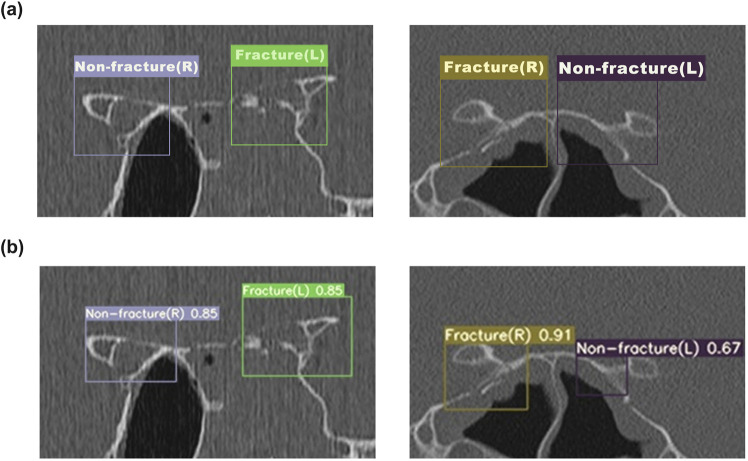
The detection results of YOLOv7 for OCF **(a)** The annotated ground truth of the optic canal region. **(b)** The predicted results of the YOLOv7 model for the optic canal region.

Figure 4 illustrates the performance of the YOLOv7 network model in identifying optic canal CT images. Five sample images were randomly selected from the optic canal CT test set. In Figure 4a denotes the annotated regions and categories of the optic canal, while Figure 4b presents the corresponding predicted results of the model, indicating the predicted lesion regions, categories, and confidence scores. Upon comparison of Figures 4a,b, our model predicts regions close to the actual lesions, demonstrating relative accuracy in detecting lesion areas.

### 3.2 Comparison of diagnostic abilities between ophthalmic resident physicians and artificial intelligence models

**FIGURE 5:**
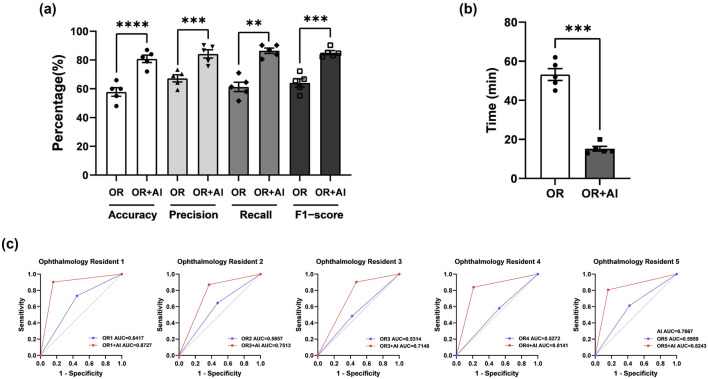
The AI model effectively assists ophthalmology residents in diagnosing OCF **(a)** Statistical analysis of Accuracy, Precision, Recall, and F-score for ophthalmology residents before and after using the AI model for assistance. Results are expressed as mean ± SEM (**: p < 0.01; ***: p < 0.001; ****: p < 0.0001). **(b)** Diagnostic duration for ophthalmology residents before and after using the AI model for assistance. Results are expressed as mean ± SEM (***: p < 0.001). **(c)** AUC-ROC values for each ophthalmology resident before and after using the AI model for assistance. The blue line represents ophthalmology residents before referring to AI, while the red line represents them after referring to AI. AUC = area under the curve; ROC = receiver operating characteristic.

We additionally selected two sets of CT images as Test Set 1 and Test Set 2 (each consisting of 50 optic canal CT images). The CT images in Test Set 1 and 2 are all from patients with unilateral OCF. We first evaluated the diagnostic abilities of five ophthalmology residents (Residents 1–5) using Test Set 1. We calculated the accuracy, precision, recall, and F1 score for each ophthalmology resident’s diagnosis (Table 4). Subsequently, we assessed the AI model’s diagnostic assistance using Test Set 2. After AI identification of the images, the diagnostic results were annotated in the upper right corner of the images, and ophthalmology residents were asked to further diagnose the annotated images. Following the reference to the AI diagnostic results, there was a significant improvement in all metrics used to evaluate the residents’ diagnostic abilities (Table 4), with statistical analysis results shown in Figure 5a. Additionally, there was a significant improvement in the diagnostic speed of ophthalmology residents, with a noticeable reduction in the total time taken to diagnose 50^−ΔΔCT^ scans (Table 4). The results of the statistical analysis are shown in Figure 5b. The ROC curve and AUC-ROC values displayed in Figure 5c exhibit a similar trend. With AI model assistance, the diagnostic capabilities of each ophthalmology resident significantly improved. Before AI assistance, the average AUC-ROC of the AI ophthalmology residents was 0.5764 ± 0.0192. After referring to the AI diagnostic results, the average AUC-ROC of the ophthalmology residents increased to 0.7954 ± 0.0271, indicating that inexperienced ophthalmology residents can make more accurate diagnoses with AI model assistance.

**TABLE 4:** Comparison of ophthalmology residents’ diagnostic capabilities before and after referring to AI.

Evaluation metric	Ophthalmology residents’ results									
	Before referring to Al results					After referring to Al results				
	OR1	OR2	OR3	OR4	OR5	OR1	OR2	OR3	OR4	OR5
Accuracy	0.66	0.6	0.48	0.55	0.6	0.87	0.79	0.72	0.82	0.84
Precision	0.733	0.69	0.593	0.643	0.703	0.903	0.794	0.757	0.866	0.892
Recall	0.71	0.645	0.516	0.581	0.613	0.903	0.871	0.903	0.839	0.806
F1-score	0.721	0.667	0.552	0.610	0.655	0.903	0.831	0.824	0.852	0.847
Time (min)	52	62	49	45	58	14	20	13	15	14

### 3.3 Evaluation of the improved UNet network for orbital apex anatomy segmentation

We improved the basic structure of the UNet network to train it for segmenting important anatomical structures in the orbital apex, including the optic canal, internal carotid artery (ICA), and sphenoid sinus. We demonstrated the superiority of the improved model from both component and overall perspectives, evaluating each component of the model through ablation experiments (Table 5). The mIoU and mDice reflect the overall segmentation performance; IoU-OC and Dice-OC are segmentation metrics for the optic canal region; IoU-ICA and Dice-ICA are metrics for the ICA region; IoU-SS and Dice-SS are metrics for the sphenoid sinus region. To enhance its ability to extract detailed textural features, we first introduced the SK Conv module independently, which increased mIoU from 0.871 to 0.9194. To address the limited receptive field problem, we attempted to introduce the TEB module alone, increasing mIoU to 0.8988. When both modules were introduced simultaneously, mIoU increased to 0.9276, which was an improvement of 0.0082 and 0.0288 compared to introducing SK Conv and TEB alone, respectively. Regarding the recognition of the optic canal region, the improved model increased IoU by 0.129 and Dice by 0.3147. For the ICA region, the improved model increased IoU by 0.0145 and Dice by 0.0241. For the sphenoid sinus region, the improved model increased IoU by 0.076 and Dice by 0.1284.

**TABLE 5:** Results of ablation experiments.

Category	mIoU	IoU-OC	IoU-ICA	IoU-SS	mDice	Dice-OC	Dice-ICA	Dice-SS
UNet	0.871	0.7505	0.8884	0.8544	0.7832	0.5151	0.8453	0.7829
UNet +SK Conv	0.9194	0.8584	0.893	0.9291	0.8875	0.7907	0.853	0.9093
UNet + TEB	0.8988	0.8754	0.9053	0.8255	0.8512	0.8225	0.8732	0.7217
UNet +SK Conv +TEB	0.9276	0.8795	0.9029	0.9304	0.9019	0.8298	0.8694	0.9113

**FIGURE 6:**
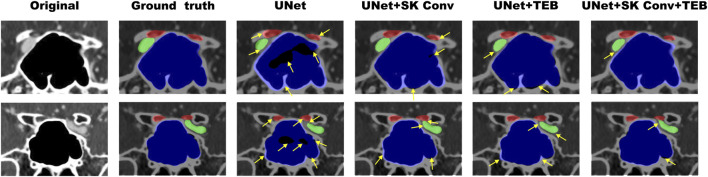
Examples of the segmentation results of enhanced orbital CT images from the ablation study. The red region represents the optic canal area, the green region represents the internal carotid artery area, and the blue region represents the sphenoid sinus area. Original refers to the original CT images, while Ground Truth represents the actual segmentation. The yellow arrows indicate areas where the AI model’s segmentation results do not match the actual conditions.

Figure 6 illustrates representative examples of the segmentation results. The black area represents the background, the red area represents the optic canal region, the green area represents the internal carotid artery region, and the blue area represents the sphenoid sinus region. From Figure 6, it can be observed that the proposed method can better reflect the real situation and achieve good segmentation for all three different anatomical regions. The original UNet network incorrectly segmented some parts of the sphenoid sinus and internal carotid artery regions as background, with overlapping edges. The addition of the SK Conv module improved this but still had some gap compared to the Ground Truth; UNet + TEB performed poorly in extracting detailed textural features. After introducing both the SK Conv and TEB modules, the segmentation results are more accurate and aligned with actual conditions. The yellow arrows indicate areas where the AI model’s segmentation results do not match the actual conditions. These results validate the necessity and superiority of the two introduced modules, demonstrating that the proposed model has significantly improved segmentation ability for the orbital apex anatomy compared to the original UNet network.

## 4 Discussion

In the realm of computer vision, four fundamental tasks prevail: image classification, object detection, semantic segmentation, and instance segmentation. Accurately selecting the appropriate task type is paramount to optimizing both accuracy and efficiency in image recognition (Tian et al., 2022). Semantic segmentation entails pixel-level classification, assigning a category label to each pixel in an image to finely delineate specific categories and detect boundaries. In this study, given the distinctive features of enhanced orbital CT images, we opted for the task of semantic segmentation. We accomplished this by training and refining the UNet network to identify specific anatomical structures within the orbital apex. UNet stands as a widely adopted deep learning convolutional neural network model tailored for image segmentation. Its architecture encompasses a compression path for spatial information capture, an expansion path for image reconstruction, and skip connections that bridge low-level features. The unique skip connections of UNet effectively capture both local and global features, ensuring segmentation accuracy, rendering it particularly adept at segmenting intricate anatomical structures in medical images (Xu et al., 2023b). Moreover, UNet demonstrates adaptability to images of varying resolutions and dimensions, rendering it suitable for the diverse datasets commonly encountered in medical imaging (Suri et al., 2023; Siddique et al., 2021; Das and Das, 2023). However, owing to inadequate consideration of the semantic gap between encoding and decoding stages and susceptibility to overfitting with limited data, UNet often encounters challenges in precisely delineating fuzzy boundaries or intricate anatomical regions (ST-Unet et al., 2023). Consequently, while the foundational UNet architecture is robust, practical applications often necessitate enhancements to accommodate diverse medical image recognition tasks.

Recent advancements in ophthalmic AI, as highlighted by Gong et al. , emphasize the critical role of tailored algorithmic improvements and multidisciplinary collaboration in addressing such challenges (Gong et al., 2024). To enhance the original UNet network’s capability to extract richer semantic information, we implemented several improvements. Firstly, we augmented its ability to extract detailed texture features by integrating a texture enhancement module based on SKNet into the first three skip connections of the UNet architecture. In recent years, SKNet has found increasing application in medical image recognition. For instance, a study by Jeny et al., in 2020 introduced a convolutional neural network model based on SKNet, which effectively classified various types of skin cancers (Jeny et al., 2020). Similarly, Cui et al. employed a 3D UNet with an SKNet attention module to achieve tooth segmentation in cone-beam computed tomography images (Cui et al., 2022). Despite these advancements, it is noteworthy that SKNet has not been extensively utilized in the realm of orbital image recognition.

The fundamental concept of SKNet revolves around grouping features and weighting these features within each group using an adaptive gating function. This adaptive adjustment mechanism enables the network to automatically leverage information captured by effective receptive fields for classification tasks. By employing SoftMax for fusion, features from kernels of different sizes are aggregated to acquire a global and comprehensive representation for weight selection. In our module, we employed four convolutional operations with varying k-sizes to extract features. Subsequently, an FFC structure computes attention scores post feature addition, facilitating attention-weighted fusion of multiple branches. This augmentation significantly enhances the accuracy and efficiency of semantic segmentation, thereby contributing to a more detailed and comprehensive understanding of the intricate anatomical structures within the orbital region.

Due to the inherent limitation of convolutional operations in establishing long-range dependencies and global contextual connections, methods based on convolutional neural networks often struggle to capture comprehensive spatial relationships (Azad et al., 2022). To address this challenge, in the last two skip connections of the UNet architecture, we introduced a semantic perception module constructed with a Transformer Encoder Block. Transformers, originally prominent in natural language processing (NLP), were ingeniously adapted to computer vision by Dosovitskiy et al., in 2021, resulting in the Vision Transformer (ViT) (Dosovitskiy et al., 2020). ViT partitions input images into smaller blocks, transforms each block into a vector, and concatenates these vectors to form a sequence. The pivotal component of ViT is a multi-layer Transformer encoder, comprising a multi-head self-attention mechanism and a fully connected feed-forward network in each encoder. The model’s output is subsequently processed by a classifier. While convolutional layers excel in local operations, focusing on relationships between adjacent pixels, Transformer layers with global operations complement convolutional networks by capturing relationships between all pixels. Transformers have been increasingly adopted in medical image segmentation studies, serving as potent encoders for various medical image segmentation tasks. Integrating Transformer with UNet facilitates the restoration of local spatial information, thereby enhancing finer details. Successful applications like TransUNet, which amalgamates the strengths of Transformer and UNet, have demonstrated impressive performance across diverse medical segmentation tasks (Yin et al., 2022). Azad et al. also combined both methods for skin lesion segmentation and achieved promising results (Azad et al., 2022).

In this study, the UNet model was enhanced to better tackle the challenges of segmenting complex anatomical structures in the orbital apex, such as the optic canal, internal carotid artery, and sphenoid sinus. To achieve this, we incorporated SKConv and TEB into the UNet architecture. These additions significantly improved the model’s ability to capture richer semantic information and overcome limitations such as the semantic gap between encoding and decoding stages, making UNet more effective at handling the detailed structures found in CT images.

By seamlessly integrating the TEB into the UNet architecture, we achieved a notable improvement in segmentation accuracy, addressing the shortcomings of traditional convolutional networks. To avoid potential loss of detailed information and image blurring—especially when processing larger images—we strategically applied the TEB only in the final two stages, ensuring maximum effectiveness while maintaining computational efficiency. The successful application of the TEB in orbital CT segmentation highlights its potential to advance medical image analysis. The results of our ablation study demonstrated the significant impact of these innovations on the model’s performance. Compared to the original UNet network, the new model’s Intersection over Union (IoU) increased from 0.871 to 0.9276, and the Dice coefficient improved from 0.7832 to 0.9019, indicating a high degree of agreement with manual segmentation results. These improvements underscore the effectiveness of our enhanced UNet model in increasing segmentation accuracy and overall performance.

The small, irregular shape and significant variability in the optic canal region make accurately defining fracture boundaries in OCF CT images a challenging task. Object detection, which can precisely identify and locate fractures while providing bounding box information, offers a clear advantage in this scenario by enabling accurate localization and size determination. To address these challenges, we selected YOLOv7 for the automatic recognition of optic canal fractures. YOLOv7 excels in real-time object detection, providing both the speed and precision necessary for initial fracture detection, especially in clinical settings where timely diagnosis is critical. Its ability to detect multiple objects in a single pass and generate precise bounding boxes ensures accurate fracture localization, even in the complex and irregular anatomy of the optic canal. In this study, after training, YOLOv7 achieved an accuracy of 0.795 and an overall sensitivity of 0.743. We also evaluated the diagnostic capabilities of ophthalmology residents before and after using YOLOv7. The average AUC-ROC increased from 0.5764 ± 0.0192 to 0.7954 ± 0.0271, while diagnostic time decreased from 68 min to 15 min. These results demonstrate that the model significantly aids ophthalmology residents, particularly those with limited experience, in quickly and accurately assessing fractures in the optic canal.

Although the YOLOv7 model demonstrated good accuracy and sensitivity in detecting optic canal fractures, there are still some limitations. False negatives may occur, especially when fractures are minor or located in difficult-to-observe areas of the optic canal, or when the fracture line is obscured due to poor image quality, such as low resolution scans or artifacts. To address these issues, we plan to expand the dataset by incorporating axial and sagittal CT images, which will provide a more comprehensive view of the optic canal and reduce missed detections. Additionally, we aim to include a wider variety of fracture types, image qualities, and patient populations to improve the robustness of the model.

Furthermore, while the improved UNet network can accurately identify anatomical structures like the internal carotid artery, optic canal, and sphenoid sinus on 2D CT images, it currently struggles to reflect the 3D relationships between the internal carotid artery and optic nerve on the sphenoid sinus wall. In future research, we will supplement the dataset with 3D CT images for better anatomical representation and integrate 3D skeletal reconstruction, which will enhance preoperative assessments and support computer-assisted surgical navigation systems. Despite these limitations, the model’s performance is still significantly better than that of less experienced ophthalmology residents, demonstrating its potential as a valuable tool for clinical auxiliary diagnosis.

## 5 Conclusion

Our study applied two image recognition tasks, namely, object detection and image segmentation, to propose an AI-assisted image recognition system for aiding in OCF diagnosis and identifying orbital apex anatomical structures. This system efficiently segments crucial anatomical structures in the orbital apex, accurately displaying anatomical details that may be overlooked by human judgment in CT scans. Importantly, the system holds promise for integration with existing navigation technologies, paving the way for a new generation of medical intelligent voice navigation systems, further enhancing the intelligence of eye and nose disease surgeries and laying the technological foundation for navigated endoscopic robotic surgeries. Moreover, the success of this specific application inspires broader medical image recognition tasks. The deployment of these models not only advances our understanding of orbital anatomy but also lays the groundwork for the continued development of AI-assisted diagnosis, promising to improve accuracy and effectiveness across various medical image recognition domains. Insights gained from refining these models pave the way for continual advancements in AI-driven diagnostics, indicating a key role for future technologies in enhancing accuracy, efficiency, and overall healthcare outcomes in various medical imaging fields.

## Data Availability

The original contributions presented in the study are included in the article/supplementary material, further inquiries can be directed to the corresponding author.

## Abbreviations

OCF, Optic canal fractures; TON, Traumatic Optic Neuropathy; RGC, Retinal Ganglion Cell; CT, Computed Tomography; AI, Artificial Intelligence; CNNs, Convolutional Neural Networks; ML, Machine Learning; DL, Deep Learning; SK Conv, Selective Kernel Convolution; TEB, Transformer Encoder Block; AP, Average Precision; AUC, Area Under the Curve; ROC, Receiver Operating Characteristic; IoU, Intersection over Union

## References

[B1] Abuzayed B. Tanriover N. Gazioglu N. Eraslan B. S. Akar Z. (2009). Endoscopic endonasal approach to the orbital apex and medial orbital wall: anatomic study and clinical applications. J. Craniofacial Surg. 20 (5), 1594–1600. 10.1097/SCS.0b013e3181b0dc23 19816303

[B2] Ahuja A. S. Wagner I. V. Dorairaj S. Checo L. Hulzen R. T. (2022). Artificial intelligence in ophthalmology: a multidisciplinary approach. Integr. Med. Res. 11 (4), 100888. 10.1016/j.imr.2022.100888 36212633 PMC9539781

[B3] Asal N. Bayar Muluk N. Inal M. Şahan M. H. Doğan A. Arıkan O. K. (2019). Carotid canal and optic canal at sphenoid sinus. Neurosurg. Rev. 42 (2), 519–529. 10.1007/s10143-018-0995-4 29926302

[B4] Azad R. Heidari M. Wu Y. Merhof D. (2022). “Contextual attention network: transformer meets U-net,” in, 13th international workshop on machine learning in medical imaging (MLMI), Singapore, SINGAPORE, vol. 13583, pp. 377–386. 10.1007/978-3-031-21014-3_39

[B5] Bi S. W. Lin H. T. Zhang K. Yang H. S. (2019). Artificial Intelligence: a framework that can distinguish cavernous hemangioma and neurilemmoma automatically. Investigative Ophthalmol. and Vis. Sci. 60 (9). http://WOS:000488628103244.

[B6] Cui W. Wang Y. Zhang Q. Zhou H. Song D. Zuo X. (2022). “CTooth: a fully annotated 3D dataset and benchmark for tooth volume segmentation on cone beam computed tomography images,” in 15th international conference on intelligent robotics and applications (ICIRA) - smart robotics for society (Harbin: PEOPLES R CHINA), 13458, 191–200. 10.1007/978-3-031-13841-6_18

[B7] Dallan I. Castelnuovo P. de Notaris M. Sellari-Franceschini S. Lenzi R. Turri-Zanoni M. (2013). Endoscopic endonasal anatomy of superior orbital fissure and orbital apex regions: critical considerations for clinical applications. Eur. Archives Oto-Rhino-Laryngology 270 (5), 1643–1649. 10.1007/s00405-012-2281-3 23179940

[B8] Das M. N. Das D. S. (2023). Attention-UNet architectures with pretrained backbones for multi-class cardiac MR image segmentation. Curr. Prob. Cardiol. 49 (1), 102129. 10.1016/j.cpcardiol.2023.102129 37866419

[B9] Dosovitskiy A. Beyer L. Kolesnikov A. Weissenborn D. Houlsby N. (2020). “An image is worth 16x16 words: transformers for image recognition at scale,” Arxiv: 2010.11929.

[B10] Gong D. Li W. T. Li X. M. Wan C. Zhou Y. J. Wang S. J. (2024). Development and research status of intelligent ophthalmology in China. Int. J. Ophthalmol. 17 (12), 2308–2315. 10.18240/ijo.2024.12.20 39697896 PMC11589450

[B11] Hamwood J. Schmutz B. Collins M. J. Allenby M. C. Alonso-Caneiro D. (2021). A deep learning method for automatic segmentation of the bony orbit in MRI and CT images. Sci. Rep. 11 (1), 13693. 10.1038/s41598-021-93227-3 34211081 PMC8249400

[B12] Hokazono Y. Umezawa H. Kurokawa Y. Ogawa R. (2019). Optic canal decompression with a lateral approach for optic nerve injury associated with traumatic optic canal fracture. Plastic Reconstr. Surgery-Global Open 7 (10), e2489. 10.1097/GOX.0000000000002489 PMC6846323 31772908

[B13] Horiguchi K. Murai H. Hasegawa Y. Mine S. Yamakami I. Saeki N. (2010). Endoscopic endonasal trans-sphenoidal optic nerve decompression for traumatic optic neuropathy -technical note. Neurol. Medico-Chirurgica 50 (6), 518–522. 10.2176/nmc.50.518 20587985

[B14] Jeny A. A. Sakib A. N. M. Junayed M. S. Lima K. A. Ahmed I. Islam M. B. (2020). “SkNet: a convolutional neural networks based classification approach for skin cancer classes,” in 23rd international conference on computer and information technology (ICCIT), ahsanullah univ sci and technol, ELECTR NETWORK. Available online at: https://ieeexplore.ieee.org/abstract/document/9392716/.

[B15] Leong Y.-Y. Vasseneix C. Finkelstein M. T. Milea D. Najjar R. P. (2022). Artificial intelligence meets neuro-ophthalmology. Asia-Pacific J. Ophthalmol. 11 (2), 111–125. 10.1097/APO.0000000000000512 35533331

[B16] Li L. Song X. Guo Y. Liu Y. Sun R. Zou H. (2020). Deep convolutional neural networks for automatic detection of orbital blowout fractures. J. Craniofacial Surg. 31 (2), 400–403. 10.1097/SCS.0000000000006069 31842071

[B17] Li X. Guo Z. (2022). Affection of surgical decompressive scale of optic canal to traumatic optic neuropathy. Brain Sci. 12 (11), 1442. 10.3390/brainsci12111442 36358368 PMC9688892

[B18] Lin J. Hu W. Wu Q. Zhang J. Yan W. (2021). An evolving perspective of endoscopic transnasal optic canal decompression for traumatic optic neuropathy in clinic. Neurosurg. Rev. 44 (1), 19–27. 10.1007/s10143-019-01208-y 31758337

[B19] Liu X. Wang J. Zhang W. Li L. Zhang L. Xiao C. (2023). Prognostic factors of traumatic optic neuropathy based on multimodal analysis-Especially the influence of postoperative dressing change and optic nerve blood supply on prognosis. Front. Neurology 14, 1114384. 10.3389/fneur.2023.1114384 PMC9922895 36793493

[B20] Ma Y.-J. Yu B. Tu Y. H. Mao B. X. Yu X. Y. Wu W. C. (2018). Prognostic factors of trans-ethmosphenoid optic canal decompression for indirect traumatic optic neuropathy. Int. J. Ophthalmol. 11 (7), 1222–1226. 10.18240/ijo.2018.07.24 30046543 PMC6048333

[B21] Nagasao T. Morotomi T. Kuriyama M. Tamai M. Sakamoto Y. Takano N. (2018). Biomechanical analysis of likelihood of optic canal damage in peri-orbital fracture. Comput. Assist. Surg. 23 (1), 1–7. 10.1080/24699322.2018.1460401 29621890

[B22] Nanegrungsunk O. Ruamviboonsuk P. Grzybowski A. (2022). Prospective studies on artificial intelligence (AI)-based diabetic retinopathy screening. Ann. Transl. Med. 10 (24), 1297. 10.21037/atm-2022-71 36660630 PMC9843399

[B23] Peng A. Li Y. Hu P. Wang Q. (2011). Endoscopic optic nerve decompression for traumatic optic neuropathy in children. Int. J. Pediatr. Otorhinolaryngology 75 (8), 992–998. 10.1016/j.ijporl.2011.05.004 21621861

[B24] Reddy R. P. Bodanapally U. K. Shanmuganathan K. Van der Byl G. Dreizin D. Katzman L. (2015). Traumatic optic neuropathy: facial CT findings affecting visual acuity. Emerg. Radiol. 22 (4), 351–356. 10.1007/s10140-014-1292-3 25563705

[B25] Siddique N. Paheding S. Elkin C. P. Devabhaktuni V. (2021). U-net and its variants for medical image segmentation: a review of theory and applications. Ieee Access 9, 82031–82057. 10.1109/access.2021.3086020

[B26] Song X. Liu Z. Li L. Gao Z. Fan X. Zhai G. (2021). Artificial intelligence CT screening model for thyroid-associated ophthalmopathy and tests under clinical conditions. Int. J. Comput. Assisted Radiology Surg. 16 (2), 323–330. 10.1007/s11548-020-02281-1 33146848

[B27] Steinsapir K. D. Goldberg R. A. (2011). Traumatic optic neuropathy: an evolving understanding. Am. J. Ophthalmol. 151 (6), 928–933. 10.1016/j.ajo.2011.02.007 21529765

[B28] ST-Unet Ye Q. Ruan T. (2023). ST-unet: swin transformer boosted U-net with cross-layer feature enhancement for medical image segmentation. Comput. Biol. Med. 153, 106516. 10.1016/j.compbiomed.2022.106516 36628914

[B29] Suri J. S. Bhagawati M. Agarwal S. Paul S. Pandey A. Gupta S. K. (2023). UNet deep learning architecture for segmentation of vascular and non-vascular images: a microscopic look at UNet components buffered with pruning, explainable artificial intelligence, and bias. Ieee Access 11, 595–645. 10.1109/access.2022.3232561

[B30] Tian D. Han Y. Wang B. Guan T. Gu H. Wei W. (2022). Review of object instance segmentation based on deep learning. J. Electron. Imaging 31 (4). 10.1117/1.Jei.31.4.041205

[B31] Ting D. S. J. Ang M. Mehta J. S. (2019). Artificial intelligence-assisted telemedicine platform for cataract screening and management: a potential model of care for global eye health. Br. J. Ophthalmol. 103 (11), 1537–1538. 10.1136/bjophthalmol-2019-315025 31481391

[B32] Trevino-Gonzalez J. L. Santos-Santillana K. M. Maldonado-Chapa F. Morales-Del Angel J. A. (2023). Neurovascular structures in the lateral recess of the sphenoid sinus. A computed tomography evaluation . Neurocir. Engl. Ed. 34 (3), 105–111. 10.1016/j.neucie.2022.11.011 36774255

[B33] Tu X. Xiong C. Qi H. Ou Y. Rao J. Sun Y. (2023). Diagnosis and treatment of transnasal endoscopic optic canal decompression for traumatic optic neuropathy. Front. Neurosci. 17, 1168962. 10.3389/fnins.2023.1168962 37260841 PMC10228362

[B34] Xu J. Shen J. Jiang Q. Wan C. Zhou F. Zhang S. (2023a). A multi-modal fundus image based auxiliary location method of lesion boundary for guiding the layout of laser spot in central serous chorioretinopathy therapy. Comput. Biol. Med. 155, 106648. 10.1016/j.compbiomed.2023.106648 36805213

[B35] Xu Y. Hou S. Wang X. Li D. Lu L. (2023b). A medical image segmentation method based on improved UNet 3+ network. Diagnostics 13 (3), 576. Art. no. 576. 10.3390/diagnostics13030576 36766681 PMC9914627

[B36] Yamashita R. Nishio M. Do R. K. G. Togashi K. (2018). Convolutional neural networks: an overview and application in radiology. Insights into Imaging 9 (4), 611–629. 10.1007/s13244-018-0639-9 29934920 PMC6108980

[B37] Yan W. Chen Y. Qian Z. Selva D. Pelaez D. Tu Y. (2017). Incidence of optic canal fracture in the traumatic optic neuropathy and its effect on the visual outcome. Br. J. Ophthalmol. 101 (3), 261–267. 10.1136/bjophthalmol-2015-308043 27267448

[B38] Yin X.-X. Sun L. Fu Y. Lu R. Zhang Y. (2022). U-Net-Based medical image segmentation. J. Health. Eng. 2022, 4189781. 10.1155/2022/4189781 PMC9033381 35463660

[B39] Yu B. Ma Y. Tu Y. Wu W. (2016). The outcome of endoscopic transethmosphenoid optic canal decompression for indirect traumatic optic neuropathy with No-Light-Perception. J. Ophthalmol. 2016, 6492858. 10.1155/2016/6492858 27965891 PMC5124648

[B40] Zhang X. Chen Y. Pu L. He Y. Zhou Y. Sun H. (2023). DASGC-unet: an attention network for accurate segmentation of liver CT images. Neural Process. Lett. 55, 12289–12308. 10.1007/s11063-023-11421-y

[B41] Zhao J. L. (2018). The development of ophthalmology in artificial intelligence era. [Zhonghua yan ke za zhi] Chin. J. Ophthalmol. 54 (9), 645–648. 10.3760/cma.j.issn.0412-4081.2018.09.002 30220177

[B42] Zhu S. J. Zhan H. D. Wu M. N. Zheng B. Liu B. Q. Zhang S. C. (2023). Research on classification method of high myopic maculopathy based on retinal fundus images and optimized ALFA-Mix active learning algorithm. Int. J. Ophthalmol. 16 (7), 995–1004. 10.18240/ijo.2023.07.01 37465510 PMC10333255

